# Photoinduced Inhibition of Neutrophil Extracellular Traps Formation by Dichromatic Light Irradiation

**DOI:** 10.3390/cimb47090729

**Published:** 2025-09-09

**Authors:** Kahramon Mamatkulov, Yersultan Arynbek, Huy Duc Le, Nina Vorobjeva, Grigory Arzumanyan

**Affiliations:** 1 Frank Laboratory of Neutron Physics, Department of Raman Spectroscopy, Joint Institute for Nuclear Research, Joliot-Curie 6, 141980 Dubna, Russia; kahramon@jinr.ru (K.M.); yersultan@jinr.ru (Y.A.); lehuy@jinr.ru (H.D.L.); 2 Faculty of Physics and Technology, Al-Farabi Kazakh National University, Al-Farabi 71, Almaty 050040, Kazakhstan; 3 Institute of Nuclear Physics, Ibragimov 1, Almaty 050032, Kazakhstan; 4 Institute of Physics, Vietnam Academy of Science and Technology, 10 Dao Tan, Hanoi 118000, Vietnam; 5 Department of Immunology, Biology Faculty, Lomonosov Moscow State University, Lenin Hills 1/12, 119234 Moscow, Russia; nvvorobjeva@mail.ru

**Keywords:** neutrophil photoactivation, cytochrome *c* oxidase, reactive oxygen species, dichromatic irradiation, photoinhibition

## Abstract

Neutrophils are the first line of defense of the human immune system against pathogens. Photobiomodulation, mediated by mitochondrial photoacceptors such as cytochrome *c* oxidase, has emerged as a method to modulate neutrophil function through targeted light exposure. Despite the extensive characterization of neutrophil extracellular traps (NETs) formation (NETosis), the wavelength-specific modulation of neutrophil photoactivation and the involvement of redox pathways remain poorly defined. In this study, the effects of monochromatic (365 nm, 415 nm, 437 nm, and 625 nm) and dichromatic LED-light irradiation on NETs formation were systematically examined. The highest netotic responses were elicited by UV-A (365 nm) and violet-blue light (415 nm), whereas 437 nm showed the lowest induction and 625 nm stimulated a moderate netotic response. The pharmacological inhibition of NETosis induced by 365 nm and 415 nm irradiation with specific NADPH oxidase inhibitor, apocynin, and mitochondrial reactive oxygen species (mtROS) scavenger, MitoTEMPO, attenuated NETs formation by engaging both enzymatic and mitochondrial oxidative sources. Notably, mtROS played a dominant role under 415 nm stimulation in contrast to 365 nm-induced NETosis as demonstrated by higher sensitivity to MitoTEMPO. Importantly, combined simultaneous irradiation with 415 nm and 625 nm LEDs resulted in a significant suppression of NETs formation by more than 50%, highlighting a potent inhibitory synergy observed for the first time and suggesting a new approach of wavelength pairing to modulate neutrophil activation. These results were further supported by measurements of ROS production using a luminol-amplified chemiluminescence assay. Collectively, these findings delineate a wavelength- and ROS-dependent framework for light-induced neutrophil activation, with mitochondrial pathways exerting central control particularly under short-wavelength irradiation.

## 1. Introduction

Neutrophil extracellular traps (NETs) formation, or NETosis, represents a specialized immune defense mechanism wherein neutrophils release web-like structures composed of decondensed chromatin and antimicrobial proteins to immobilize and neutralize pathogens [1,2]. This process is tightly regulated by redox-sensitive signaling pathways, with reactive oxygen species (ROS) serving as essential mediators for chromatin decondensation and NETs release [3,4]. Given the susceptibility of neutrophils to redox perturbations, external factors that influence intracellular redox homeostasis, such as light exposure, are likely to modulate NETosis dynamics.

The interaction of light with biological systems has long been a subject of scientific inquiry [5,6,7,8], particularly in relation to its ability to modulate cellular and biochemical processes through photoexcitation [9]. In the context of redox biology and immune function, light absorption by photoacceptor molecules can induce significant biochemical changes [10,11,12], notably influencing the redox properties of electron carriers and, consequently, cellular metabolism [13,14]. A crucial aspect of this interaction lies in the capacity of specific wavelengths to modulate the redox state of key electron transfer components, such as those in cytochrome *c* oxidase and other respiratory chain complexes [15,16]. These photobiological effects extend beyond simple energy absorption, triggering secondary molecular and cellular responses that may include ROS generation and redox-dependent signaling pathways [17,18,19].

Recent evidence suggests that variations in photon absorption across different wavelengths influence the redox state of respiratory complexes, thereby affecting oxidative stress responses and immune cell activation [20,21]. In our previous study, we explored the effect of light exposure at various wavelengths on NETs formation, which plays a pivotal role in immune defense and inflammation [22]. Specifically, our prior findings indicate that certain wavelengths may be relevant for promoting NETosis, suggesting that light may exert bidirectional regulatory effects in case of dichromatic exposure on neutrophil activity [22]. The underlying mechanism likely refers to the possibility of inner alterations within redox centers in electron transfer pathways, which subsequently modulate oxidative stress levels and inflammatory responses.

In mitochondria, four key redox centers within the electron transport chain (ETC)—Complex I (NADH: ubiquinone oxidoreductase), Complex II (succinate dehydrogenase), Complex III (cytochrome bc1 complex), and Complex IV (cytochrome *c* oxidase)—orchestrate a series of electron transfer reactions that drive ATP synthesis via oxidative phosphorylation [23,24,25]. However, these redox centers also serve as major sources of ROS, whose levels fluctuate in response to cellular metabolic demands and external stimuli, including photonic interactions. This dual role of mitochondria, balancing ATP production and ROS generation, highlights their pivotal contribution to immune cell function [26,27]. By modulating mitochondrial redox activity, light exposure may fine-tune neutrophil responses, either amplifying ROS-driven NETs formation or mitigating excessive oxidative stress, thereby influencing the overall inflammatory landscape.

Light exposure has been shown to modulate the activity of these redox centers, influencing both ROS production and ATP synthesis [28]. In particular, photon absorption by mitochondrial chromophores, such as cytochrome *c* oxidase, can modulate electron transfer efficiency, potentially leading to increased ATP production under specific photonic conditions [29,30]. Conversely, excessive excitation may lead to electron leakage and augmented ROS generation, which can trigger oxidative stress and cellular signaling cascades [31]. Photon absorption, particularly in the visible and near-infrared spectrum, has been demonstrated to alter cytochrome *c* activity, potentially enhancing its electron transfer efficiency and thereby increasing ATP synthesis [18].

In the context of immune cell activation, especially NETosis, ROS play a crucial role in initiating and sustaining the process [32,33]. Elevated ROS levels act as secondary messengers, triggering pathways that promote chromatin decondensation and NETs formation [4,32,34,35,36].

At present, there are only a small number of publications concerning the effect of monochromatic irradiation on NETs formation [37,38,39,40,41,42]. However, a number of studies [43,44,45] led us to suggest that dichromatic irradiation may have specific pathways of action on biological responses of cells as compared with ordinary monochromatic irradiation. In particular, as demonstrated in [43], the simultaneous irradiation of HeLa cells at specific wavelengths resulted in a modification in the ratio of the reduced and oxidative forms of cytochrome *c* oxidase. This, in turn, affected cell survival. However, the application of dichromatic irradiation to NETosis research has not been previously explored. The present study therefore aimed to systematically investigate the effects of monochromatic and dichromatic light irradiation at specific wavelengths on NETosis and the oxidative burst of human neutrophils.

## 2. Materials and Methods

### 2.1. Reagents

MitoTEMPO (mtROS inhibitor), DAPI (DNA dye), and phorbol 12-myristate 13-acetate (PMA, protein kinase C activator) were procured from Sigma-Aldrich (St. Louis, MO, USA). Apocynin (NADPH oxidase inhibitor) was obtained from Abcam (Cambridge, UK). The Ficoll-Hypaque with densities of 1.119 and 1.077 g/cm^3^ and RPMI1640 medium containing L-glutamine were purchased from PanEco Ltd. (Moscow, Russia). Luminol was purchased from JSC LenReactive (Moscow, Russia).

### 2.2. Isolation of Primary Human Neutrophils

The venous blood samples were obtained from healthy adult volunteers under rigorous ethical oversight in strict accordance with the guidelines set by the Ethical Committee of the Faculty of Biology, Lomonosov Moscow State University. Prior to participation, all individuals were provided with comprehensive information regarding the study’s objectives, procedures, and potential risks, in line with the World Health Organization (WHO) standards for biomedical research involving human subjects, as delineated in the WHO Handbook for Good Clinical Research Practice (GCP) and aligned with the ethical framework established by the Declaration of Helsinki (2013 revision, Fortaleza, Brazil). Three of the authors contributing to this study—H.L.D., Y.A., and K.M.—voluntarily participated as donors. Their inclusion ensured immediate availability of fresh samples and permitted precise procedural adherence throughout the experimental process.

Peripheral blood was collected in EDTA-coated tubes (Minimed, Bryansk Region, Russia) and diluted with phosphate-buffered saline (PBS, pH 7.4) at a blood to PBS ratio of 2:1. Neutrophils were isolated by double-density gradient centrifugation using Ficoll-Hypaque (1.077 and 1.119 g/cm^3^) at 400 g for 40 min and room temperature (RT) as described previously [22]. After thorough washing with PBS, contaminating erythrocytes were lysed in bidistilled water for 1 min. Isotonicity was then restored with concentrated PBS. Isolated neutrophils were suspended in RPMI1640 to a concentration of 2 × 10^5^ cells/mL. The choice of a medium free of light-sensitive substances, such as serum, prevents the occurrence of unwanted cellular reactions induced by light. Microscopic examination of the isolated cells showed that more than 97% of them were neutrophils. Cell viability, determined by trypan blue exclusion, was more than 98%.

### 2.3. Experimental Design and Light Irradiation Protocol

Freshly isolated neutrophils were irradiated with LEDs purchased from Shenzhen Grace Optoelectronics Co., Ltd., Shenzhen, China. The irradiation dose for all wavelengths was standardized to 32 J/cm^2^, calculated as the power in joules per exposed area, with a total exposure time of 6 min. The irradiation power of the LEDs was measured using a PM100A power meter (Thorlabs, Newton, NJ, USA).

**Figure 1:**
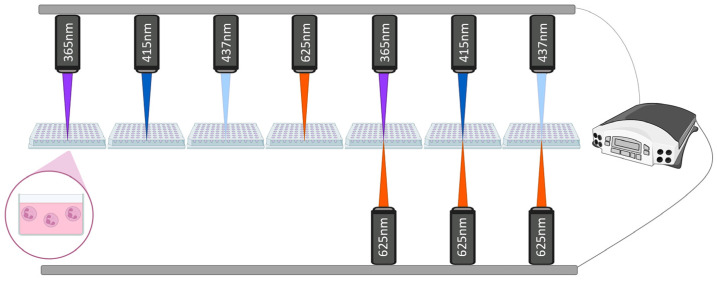
Schematic illustration of mono- and dichromatic irradiation of neutrophils. Freely isolated neutrophils (2 × 10^5^ cells/mL) were seeded into the wells of a 96-well plate in RPMI1640 medium for 30 min at 37 °C and 5% CO_2_ for adhesion. For monochromatic irradiation, neutrophils were exposed separately to specific wavelengths of 365 nm, 415 nm, 437 nm, and 625 nm. In dichromatic irradiation protocols, two-wavelength combinations were applied simultaneously and included the following pairs: (365 + 625) nm, (415 + 625) nm, and (437 + 625) nm. All light exposure protocols utilized a calibrated irradiation system with an upper-positioned light source, and for simultaneous dichromatic exposure, an additional lower light source was employed.

Neutrophils (2 × 10^5^ cells/mL) were seeded into the wells of a 96-well plate in RPMI1640 medium for 30 min at 37 °C and 5% CO_2_ for adhesion. For monochromatic irradiation, neutrophils were exposed separately to specific wavelengths of 365 nm, 415 nm, 437 nm, or 625 nm. In dichromatic irradiation protocols, two-wavelength combinations were applied simultaneously and included the following pairs: (365 + 625) nm, (415 + 625) nm, and (437 + 625) nm. All light exposure protocols utilized a calibrated irradiation system with an upper-positioned light source and, for simultaneous dichromatic exposure, an additional lower light source was employed (Figure 1).

Where indicated, neutrophils were preincubated for 30 min with 400 μM apocynin or for 40 min with 25 μM MitoTEMPO.

### 2.4. Assessment of NETosis with Fluorescence Microscopy

Following a two-hour incubation, irradiated and non-irradiated (control) neutrophils were fixed with 4% paraformaldehyde for 30 min, after which they were washed twice with PBS in order to remove any residual medium or unbound substances [46]. The cells were then stained with DAPI at a final concentration of 10 µM for 10 min in the dark and RT to specifically label DNA. After the staining, cells underwent PBS washing to eliminate excess dye. The micro-images were obtained with a Nikon Eclipse Ts2R-FL fluorescent LED microscope, which was fitted with NIS-Elements BR software (version 5.30.05), an Epi-FL C-LED385 filter, and a CFI Super Plan Fluor ELWD ADM20 objective with a numerical aperture (NA) of 0.45 and a working distance ranging from 8.2 to 6.9 mm. A total of 20 frames were acquired per well to ensure adequate sampling. The collected images were analyzed using ImageJ software (version 1.53q) to quantify the number of netotic and intact cells across the 20 frames, allowing for a comprehensive evaluation of NETosis.

Netotic neutrophils were defined as cells with extracellularly localized chromatin as described previously [22]. Total cell numbers and netotic cell numbers were counted in multiple fields of view, and the percentage of neutrophils forming netotic neutrophils was calculated. For each wavelength and wavelength combination, five independent experiments were performed with neutrophils isolated from different donors, and approximately 500 cells were analyzed.

### 2.5. Luminol-Enhanced Chemiluminescence Assay

To detect the total amount of reactive oxygen species (intra- and extracellular), the luminol-enhanced chemiluminescence method was used. Neutrophils (2 × 10^5^ cells/mL in RPMI1640) were freshly isolated and then separately exposed to specific wavelengths of 365 nm, 415 nm, and 625 nm. For the dichromatic irradiation protocol, combinations of two wavelengths were used simultaneously including the following pairs: (365 + 625) nm and (415 + 625) nm. The chemiluminescence was recorded in 600 μL of cell suspension with the addition of 200 μM luminol for 2.5 h with one-second integration time using a 12-channel chemiluminometer Lum-1200 (DISoft Ltd., Moscow State University, Moscow, Russia).

### 2.6. Statistical Analysis

All data were analyzed using GraphPad Prism v10.4.0 (GraphPad Software, San Diego, CA, USA). A two-way analysis of variance (ANOVA) with a Tukey post hoc test was employed to identify substantial discrepancies between monochromatic irradiation and the incorporation of inhibitors such as apocynin and MitoTEMPO. The data for the release of the NETs and the kinetics of ROS formation under mono- and dichromatic irradiation were statistically compared using one-way ANOVA with Tukey’s post hoc test. The differences were considered significant at *, *p* < 0.05, **, *p* < 0.01, ***, *p* < 0.001, and ****, *p* < 0.0001.

## 3. Results

### 3.1. Effects of Monochromatic and Dichromatic Irradiation on NETs Formation

In our previous publication [22], we demonstrated for the first time that NETs formation can be induced not only by UV-A irradiation but also by visible light ranging from blue to red, using different wavelengths and doses of monochromatic irradiation. The objective of this study was to ascertain whether dichromatic LED irradiation could engender synergistic or, conversely, inhibitory effects.

**Figure 2:**
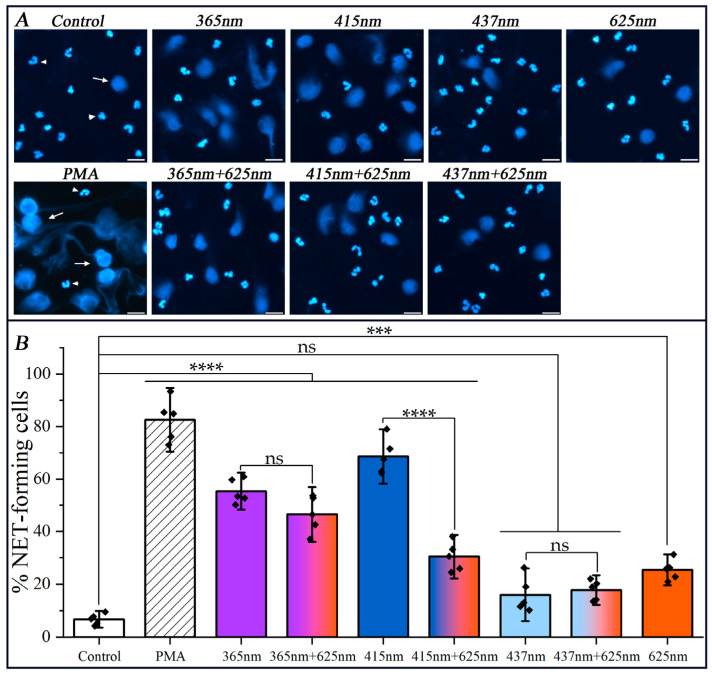
The release of NETs depending on mono- and dichromatic irradiation. Freshly isolated human neutrophils were irradiated with LED-lengths of mono- (365, 415, 437, 625 nm), or simultaneously with dichromatic wavelengths (365 + 625, 415 + 625, 437 + 625) nm at the dose of 32 J/cm^2^. The well-known protein kinase C activator PMA (50 nM) was used as a positive control. Neutrophils were incubated in RPMI1640 for 2 h at 37 °C and 5% CO_2_. The cells were fixed with 4% paraformaldehyde and stained with DAPI to visualize chromatin. (**A**) Representative fluorescence microscopy images of NETs release depending on mono- and dichromatic irradiation. NET-forming cells are indicated with arrows, and intact cells are indicated with arrow heads. Scale bars, 20 μm. Magnification, 20×. (**B**) Percentage of NETosis measured in five independent experiments with neutrophils isolated from different donors. (*n* = 5). More than 500 cells were analyzed. Data are presented as mean ± SD. The symbol (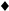) indicates the mean value from each of the five experiments. The colors are conventionally characterized by their wavelengths, and the slanted pattern refers to PMA. ns., not significant; ***, *p* < 0.001; ****, *p* < 0.0001.

The following wavelengths, 365 nm (UV-A), 415 nm (violet-blue), 437 nm (blue), 625 nm (reddish-orange), and their combinations (365 + 625) nm, (415 + 625) nm, and (437 + 625) nm, were applied in our research (Figure 1 and Figure 2). With the exception of the 437 nm LED, the selection of LEDs was adapted to correspond with the absorption spectra bands of cytochrome *c* and cytochrome *b*_558_ [47,48].

Among all tested monochromatic exposures, 415 nm irradiation triggered the most pronounced netotic response, resulting in 68.6% NETs release, followed by 365 nm with 55.4% NETs formation (Figure 2). In contrast, 437 nm and 625 nm monochromatic irradiations yielded significantly lower NETs responses, at 16.0% and 25.4%, respectively.

To verify NETs formation at 415 nm and 32 J/cm^2^, neutrophils were immunostained using FITC-conjugated monoclonal mouse anti-human MPO priming antibody and DAPI (Supplementary Figure S1).

Although 365 nm light carries higher photon energy due to its shorter wavelength, the enhanced response to 415 nm is likely attributed to its stronger absorption in the Soret band of cytochrome *c* oxidase, a known mitochondrial photoacceptor as reported in [18,49]. It is noteworthy that the Soret band of heme compounds, with a wavelength of (400–420) nm, exhibits a significantly higher intensity compared to the absorption bands within the visible range. This emphasizes that absorption efficiency may supersede photon energy in determining cellular photoreactivity. This assumption was confirmed by the observation of photoactivation of neutrophils at a wavelength of 437 nm, where both cytochromes exhibited an absence of a pronounced absorption peak. Consequently, the yield of NETs formation did not significantly exceed that of the control group, thus rendering them of minimal interest in the study of photoinhibition of NETs formation (Figure 2).

The most striking outcome was observed under simultaneous dichromatic exposure to 415 nm and 625 nm light, which led to a greater than 50% reduction in NETs formation compared to 415 nm alone (30.4% vs. 68.6%). This pronounced inhibitory effect suggests a wavelength-dependent antagonism, potentially arising from differential modulation of mitochondrial and cytosolic redox pathways or competitive binding to photoacceptors with opposing downstream signaling effects.

Other combinations, such as (365 + 625) nm and (437 + 625) nm, produced intermediate or minor effects (46.5% and 17.7%, respectively), while not exceeding the NETs-inductive potential of their most effective monochromatic component. The lack of additive or synergistic effects in these cases further underscores the specificity and complexity of wavelength interactions in shaping neutrophil activation profiles.

In summary, the formation of NETs is strongly influenced by the wavelength and spectral combination of light exposure. This is probably mediated by mitochondrial ROS generation and NADPH oxidase activity, which influence the dynamics of chromatin decondensation.

**Figure 3:**
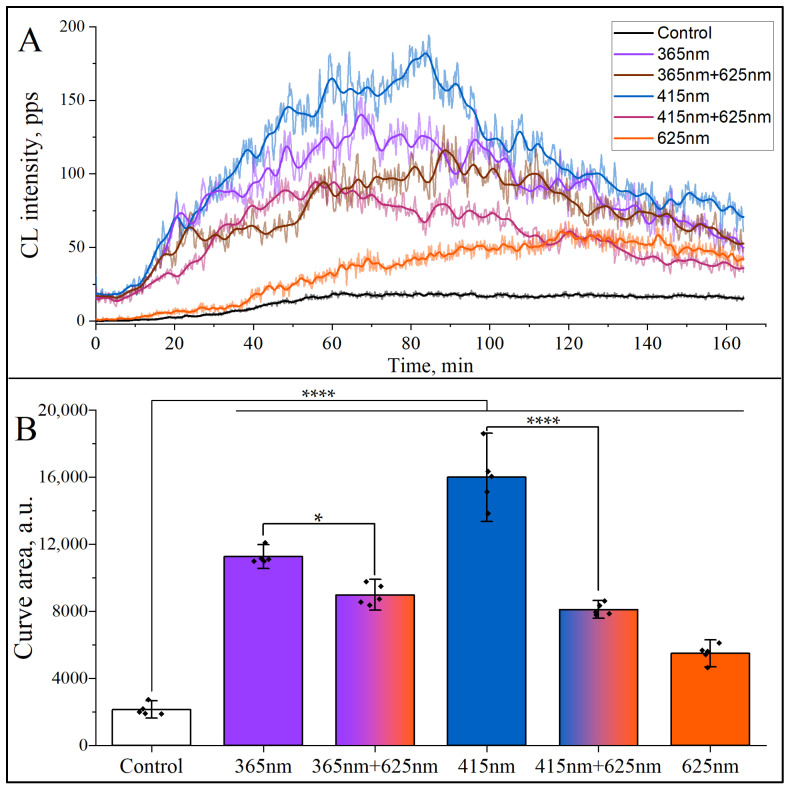
Kinetics of reactive oxygen species (ROS) formation in human neutrophils under mono- and dichromatic irradiation. Freshly isolated human neutrophils were irradiated with mono- (365, 415, and 625 nm) and dichromatic (415 + 625, and 365 + 625) nm wavelengths, and luminol-amplified chemiluminescence was recorded immediately using chemiluminometer Lum-1200. (**A**) Typical chemiluminescence kinetic curves are shown. On the abscissa axis: time, min. On the ordinate axis: chemiluminescence intensity in photons per second (pps). (**B**) Light emission induced by oxidative burst activators is expressed as an area under chemiluminescence curves. (*n* = 5). The symbol (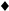) indicates the mean value from each of the five experiments. The colors are conventionally characterized by their wavelengths. Abbreviations: CL, chemiluminescence; a.u., arbitrary units; *, *p* < 0.05; ****, *p* < 0.0001.

An important component of NETosis in human neutrophils is ROS formation [1,4] in the process called the “oxidative burst”. To elucidate whether mono- and dichromatic irradiation induces ROS formation, a luminol-amplified chemiluminescence assay was used in our study. As can be seen in Figure 3, all three monochromatic wavelengths (365 nm, 415 nm, and 625 nm) and their combination, i.e., dichromatic irradiation (365 + 625, 415 + 625) nm, induced the increase in the oxidative burst in human neutrophils. It is important to note that there is a strong correlation between the data on NETosis shown in Figure 2 and the integral ROS production demonstrated in Figure 3. Interestingly, the NETosis inhibition coefficient for dichromatic irradiation (415 + 625) nm is about 2.2 in comparison to NETosis yield under mono-irradiation (415 nm), and the ratio of ROS yield for the same radiation pair is around 1.9. Similar coefficients of NETosis inhibition and ROS production were also found for another pair of mono- (365 nm) and dichromatic radiation (365 + 625) nm, i.e., 1.24 and 1.19, respectively.

### 3.2. Differential Modulation of NETosis Induced by Monochromatic Light with ROS-Targeting Inhibitors

In the second part of our study, we aimed to uncover the intracellular oxidative mechanisms underlying NETosis induced by defined monochromatic light exposures, using selective pharmacological inhibitors to analyze the redox contributions of mitochondrial versus NADPH oxidase-mediated sources. For that, the following inhibitors were used: apocynin, a well-established inhibitor of NADPH oxidase [50,51], and MitoTEMPO, which is a mitochondria-targeted antioxidant that scavenges superoxide radicals generated within the mitochondrial matrix [52,53].

**Figure 4:**
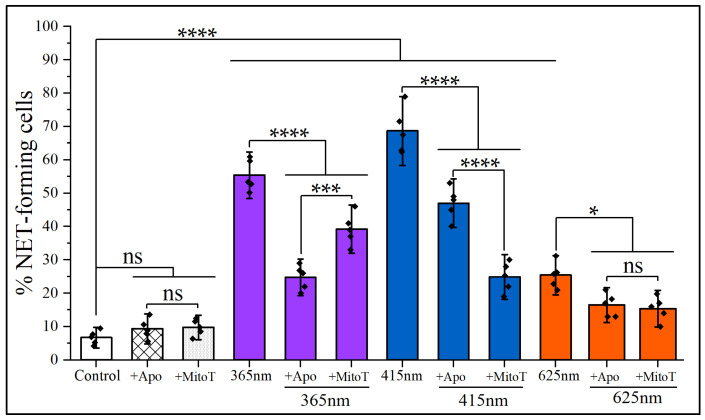
Effects of selective inhibitors of NADPH oxidase and mitochondrial ROS on NETs for-mation in non-irradited and irradiated neutrophils with three LED-sources. Neutrophils were iso-lated from healthy donors and subsequently treated with selective inhibitor of NADPH oxidase (400 μM apocynin) and mitochondrial ROS scavenger (25 μM MitoTEMPO) for a period of 30 and 40 min, respectively. Subsequently, cells were exposed to 365 nm, 415 nm, and 625 nm wavelengths, with the same energy dose of 32 J/cm^2^. NETosis was registered after 2 h incubation at 37 °C and 5% CO_2_. The data represent the mean ± SD from five independent experiments (*n* = 5). The symbol (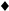) indicates the mean value from each of the five experiments. Colors are conventionally characterized by their wavelengths. The checkered and dotted patterns correspond to Apocynin and MitoTEMPO, respectively. ns, not significant; *, *p* < 0.05; ***, *p* < 0.001; ****, *p* < 0.0001.

As shown in Figure 4, the incubation of neutrophils with apocynin or MitoTEMPO before the irradiation at 365 nm substantially suppressed NETosis, indicating that both NADPH oxidase-dependent and mtROS-mediated pathways contribute to UV-A–induced neutrophil activation. Likewise, NETosis triggered by 415 nm irradiation was markedly reduced in the presence of MitoTEMPO (2.75 times) and almost twice less inhibited by apocynin, highlighting a predominant involvement of mitochondrial oxidative signaling in blue light–mediated NETs formation. In contrast, NETs formation induced by 625 nm light, although lower in magnitude, was also almost equally sensitive to both inhibitors, suggesting that red light-induced NETosis also involves ROS production, albeit through less efficient or alternative mechanisms. Notably, despite the common ROS dependence across all wavelengths, the differential inhibition patterns highlight that mitochondrial pathway may play a central and wavelength-amplified role, particularly in blue-light-mediated photostimulation. These findings delineate a complex interplay between irradiation wavelength and intracellular oxidative mechanisms in regulating neutrophil extracellular trap formation.

## 4. Discussion

The present study identifies photonic energy as a strong modulator of NETs formation through mechanisms that are both wavelength-specific and redox-dependent. Our data show that shorter wavelengths, particularly in the ultraviolet (365 nm) and violet-blue light (415 nm) regions, strongly initiate NETosis. This probably occurs through the photoactivation of intracellular chromophores such as cytochrome *c* oxidase, which absorbs maximally within the Soret band. In contrast, longer wavelengths such as 437 nm and 625 nm exhibit significantly lower efficacy.

Importantly, experiments with pharmacological suppression of NETosis using the NADPH oxidase inhibitor apocynin and mitochondria-targeted antioxidant MitoTEMPO reveal that the underlying redox signaling is highly dependent on the wavelength of irradiation. Consequently, violet-blue light-induced NETs formation is predominantly driven by mtROS, whereas ultraviolet light engages both mtROS and NADPH oxidase-mediated pathways. Even the less effective 625 nm irradiation has been shown to involve ROS, thus suggesting the presence of wavelength-tuned oxidative circuits within neutrophils.

The most intriguing aspect of our investigation concerns the effects of dichromatic light exposure. When a particular pair of wavelengths was applied simultaneously, we observed unique outcomes compared to single-wavelength exposures, hinting at antagonistic interactions at the molecular level [54]. Specifically, the study [43] demonstrated that the simultaneous dichromatic irradiation of HeLa cells resulted in a significant alteration in absorption spectrum of cytochrome *c* oxidase. These alterations are characterized by spectral shifts within the blue-violet Soret band region or even its disappearance; the appearance of new bands in the green region of the spectrum, etc. Consequently, this led to a modification in the ratio of the reduced and oxidative forms of this enzyme in one of its intermediate forms.

It was logical to assume that in the case of dichromatic irradiation of neutrophils, the results of photoactivation of NETosis would differ from those observed with monochromatic irradiation. As demonstrated in Figure 2, the simultaneous irradiation of neutrophils at 415 nm and 625 nm wavelengths leads to a significant suppression of NETs formation in comparison to single-wavelength irradiation with 415 nm exposure. The decline in NETs formation by more than 2.0 times suggests that the secondary simultaneous exposure to 625 nm exerts an inhibitory effect, potentially through modulation of intracellular oxidative stress and interference with ROS-mediated pathways. Given that red light has been linked to mitochondrial regulation, its suppressive effect may be attributed to a reduction in oxidative stress. The validity of this assumption was confirmed by the results of the chemiluminescent analysis (Figure 3). This reduction in NETs yield underscores a wavelength-dependent modulation of neutrophil activation, wherein reddish-orange light at 625 nm counteracts the pro-inflammatory response triggered by shorter wavelength violet-blue light. These findings highlight the intricate interplay between different wavelengths of light in immune cell regulation, thereby offering a potential therapeutic intervention strategy for controlling excessive neutrophil activation and inflammatory responses.

An important component of neutrophil activation is a rapid increase in ROS production, called the “oxidative burst”. The major generator of ROS in human neutrophils, addition to mitochondria, is NADPH oxidase, which is assembled from several different subunits at the plasma membrane and in the specific granules [55]. NADPH oxidase is responsible for the transfer of electrons from NADPH to molecular oxygen to form superoxide anion radicals (O_2_·^–^). O_2_·^–^ in turn spontaneously dismutates with the transformation for hydrogen peroxide (H_2_O_2_). These primary ROS can be further processed to form more reactive metabolites such as the hydroxyl radical (OH·) and hypochlorous acid (HCLO).

In our study, the ROS formation was recorded using luminol-enhanced chemiluminescence assay, and the results showed that all tested monochromatic wavelengths and their combinations induced ROS, which correlated with the release of NETs under the same LEDs (Figure 3). This correlation confirms the involvement of ROS in suicidal NETosis, which promotes the dissociation of protein complexes—azurosomes, located in azurophilic granules of neutrophils [4]. This process results in the release of serine proteases into the cytosol and their subsequent migration into the nucleus, where they participate in the decondensation of nuclear chromatin [4].

The method using luminol as a chemiluminescent enhancer was not chosen sporadically. Luminol is the most common chemiluminescent reagent, which generates chemiluminescence upon oxidation by ROS and hydrogen peroxide. In addition, luminol freely migrates through cell membranes, which allows recording the total amount of ROS, both intra- and extracellular.

We should mention that only a few scientific groups are investigating the effects of light irradiation on NETosis [37,38,39,40,41]. All these studies use only monochromatic radiation and a limited number of wavelengths. The novelty of our study lies in the use of a wide range of visible wavelengths and dichromatic irradiation of neutrophils.

Thus, by elucidating the complex interplay between light absorption, redox dynamics, and NETosis, this study contributes to a broader understanding of the photobiological regulation of immune responses. The ability of different wavelengths to either enhance or suppress neutrophil activation underscores the complexity of photoinduced biochemical modulation and opens new avenues for targeted therapeutic interventions in immune regulation and inflammatory disease management. A key aspect of this regulation lies in the redox-sensitive mechanisms governing mitochondrial activity, as mitochondria serve as central hubs for both energy metabolism and ROS production.

### Concluding Remarks

The present study constitutes pioneering work on dichromatic activation of photonetosis. Importantly, combined simultaneous irradiation with 415 nm and 625 nm LEDs resulted in a significant suppression of NETs formation by more than 50%, highlighting a potent inhibitory synergy that has not been previously observed and suggesting a new approach to wavelength pairing to modulate neutrophil activation. The findings establish a finely orchestrated relationship between light wavelength, redox biology, and innate immune function. Furthermore, the translational potential of using spectrally precise phototherapies to regulate NETosis in inflammatory and autoimmune disorders was indicated. Thus, our findings highlight a fundamental principle: photonic modulation of neutrophil activation is not solely dependent on the energy of absorbed photons but is intricately linked to cellular metabolic and redox homeostasis.

## Data Availability

The data that support the findings of this study are available on request from the authors.

## Supplementary Materials

The following supporting information can be downloaded at: https://www.mdpi.com/article/10.3390/cimb47090729/s1.
