# Supplementary material for: Photoinduced Inhibition of Neutrophil Extracellular Traps Formation by Dichromatic Light Irradiation
Source: Curr Issues Mol Biol. 2025 Sep 9;47(9):729. doi: 10.3390/cimb47090729 (PMC12468020; doi:10.3390/cimb47090729)
Supplement: Supplementary file 1 [file cimb-47-00729-s001.zip › cimb-3855560-supplementary.pdf]

Supplementary material

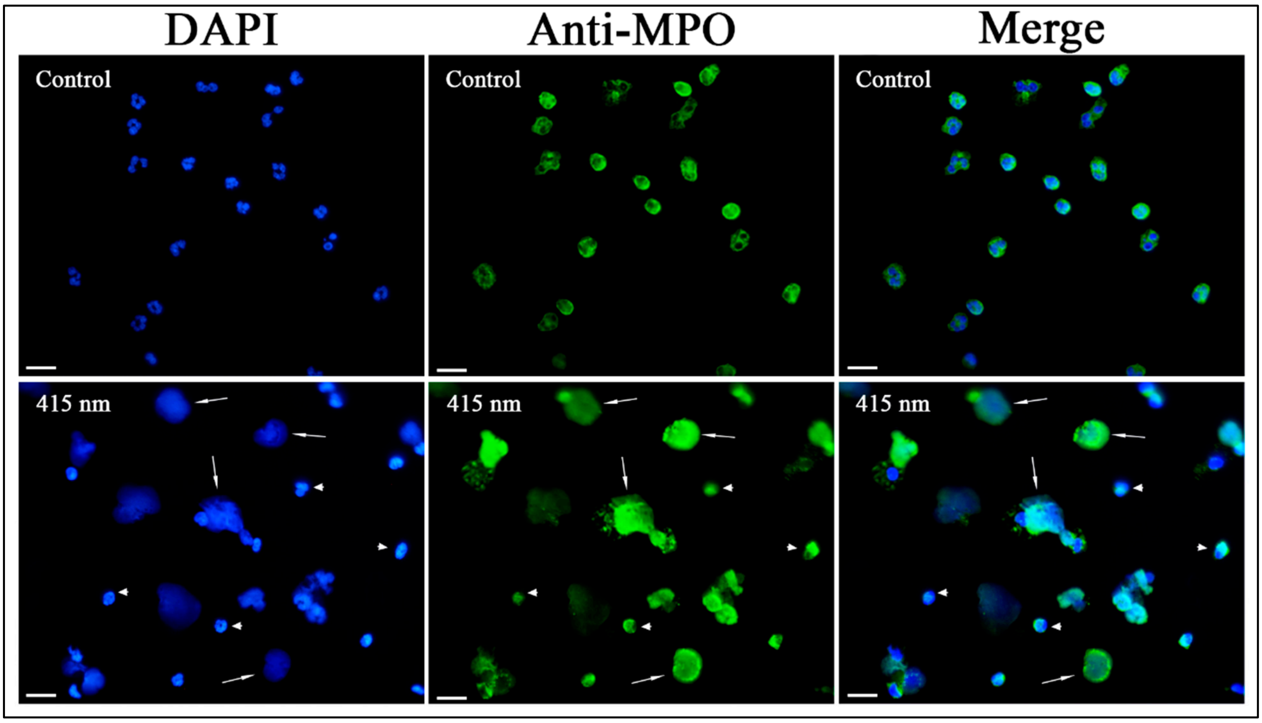

Supplementary Figure S1. Immunofluorescence images of intact and irradiated human neutrophils.

Irradiation of human neutrophils at violet-blue (415 nm wavelength) with energy dose of 32 J/cm^2 caused NETs formation. For immunofluorescence staining, neutrophils were fixed with 4% paraformaldehyde for 30 min, washed with PBS, and permeabilized with 0.1% Triton X-100 in PBS for 2 min at room temperature. Nonspecific binding was reduced by pre-incubation of cells with blocking buffer (including human immunoglobulins) for 20 min. Immunofluorescence staining was performed with FITC-conjugated monoclonal mouse anti-human MPO priming antibody (Invitrogen, USA). Blue color, staining of chromatin with DAPI. Green color, staining of MPO with FITC-conjugated anti-MPO monoclonal antibody. NETs-forming cell are indicated with arrows, and intact cells are indicated with arrow heads. Magnification, 40x. Scale bars, 25μm.
